# Supplementary material for: Nucleolar structure connects with global nuclear organization
Source: bioRxiv. 2023 Mar 31:2023.03.30.534966. Preprint. [Version 1] doi: 10.1101/2023.03.30.534966 (PMC10081344; doi:10.1101/2023.03.30.534966)
Supplement: 1 — Suppl. Table 1: * The exonic and intronic regions of the human reference genome hgp38 were searched with a criterion set at a GC content of between 65 and 100%, as detailed in Materials and Methods. NA: not annotated. Suppl. Figure 1: The transcription of rDNA was significantly reduced in cells treated with siRPA194 and to a lesser extent with siUTP4. (A) BrU pulse labeling for 5 minutes showed a significant loss of BrU signal in siRPA194 treated cells (A, arrows), compared to controls where more intense labeling was detected in nucleoli. (B) Quantitative RT-PCR showed a significant reduction in 5’ETS RNA expression in siRPA194 and siUTP4 treated cells. Bar= 5 mm. ****p<0.001, **p<0.01 Suppl. Figure 2: siRPA194, but not siUTP4 treated HeLa cells, lost the RPA194 signal (green). Nucleolar segregation was detected by the immunolabeling of fibrillarin (magenta). Cap-like structures enriched with firbrillar components (arrows) juxtaposed the reminants of the nucleoli primarily containing granular components (arrowheads). Bars= 5 mm Suppl. Figure 3: Treatment with a low concentration (0.04ug/ml) of actinomycin D (Act D) induces nucleolar segregations and disruption of Cajal bodies as detected by anti-fibrillarin antibodies (red) and anti-coilin (green) antibodies. Bar= 5 mm Suppl. Figure 4: The CRISPR-Cas9 sgRNA labeling of rDNA was validated using mitotic cells where UBF was found to be colocalized with the labeling (arrows), confirming that the sgRNA sets indeed labeled rDNA chromatin on mitotic chromosomes (arrow). Bar= 5 mm Suppl. Fig. 5: Cajal bodies are significantly altered in siRPA194 treated cells. Cells were co-immunolabeled with antibodies against UBF and coilin. Bar= 5 mm Suppl. Figure 6: While Cajal bodies change significantly in siRPA194 treated cells, histone locus bodies do not show detectable changes. Bar= 5 mm [file NIHPP2023.03.30.534966v1-supplement-1.pdf]

bioRxiv preprint doi: https://doi.org/10.1101/2023.03.30.534966; this version posted March 31, 2023. The copyright holder for this preprint (which was not certified by peer review) is the author/funder, who has granted bioRxiv a license to display the preprint in perpetuity. It is made available under aCC-BY 4.0 International license.

Supplemental material:

Suppl. Table 1: * The exonic and intronic regions of the human reference genome hgp38 were searched with a criterion set at a GC content of between 65 and 100%, as detailed in Materials and Methods. NA: not annotated.

Table S1. Human reference genome sites 65-100% GC*

ID	GC content	Symbol	Gene name
100422838	0.92857143	MIR3195	microRNA 3195
100616250	0.87912088	MIR3960	microRNA 3960
100313781	0.86842105	MIR718	microRNA 718
100616436	0.86363636	MIR4449	microRNA 4449
100616154	0.85185185	MIR4466	microRNA 4466
101929080	0.85106383	LINC02292	long intergenic non-protein coding RNA 2292
102464817	0.83636364	MIR5787	microRNA 5787
100616301	0.82758621	MIR4674	microRNA 4674
100616467	0.82051282	MIR4767	microRNA 4767
100616364	0.80821918	MIR4785	microRNA 4785
102724890	0.80	LINC02293	long intergenic non-protein coding RNA 2293
100616138	0.79761905	MIR4787	microRNA 4787
100616202	0.7962963	MIR4634	microRNA 4634
693157	0.78947368	MIR572	microRNA 572
102465978	0.78688525	MIR6850	microRNA 6850
102465503	0.77777778	MIR6836	microRNA 6836
100422962	0.77419355	MIR4281	microRNA 4281
102464833	0.77272727	MIR6084	microRNA 6084
100616288	0.7721519	MIR4665	microRNA 4665
100616143	0.77173913	MIR4763	microRNA 4763
100287284	0.77142857	MANSC4	MANSC domain containing 4
3222	0.77142857	HOXC5	homeobox C5
8408	0.77142857	ULK1	unc-51 like autophagy activating kinase 1
100616270	0.76712329	MIR4651	microRNA 4651
693149	0.76595745	MIR564	microRNA 564
100302196	0.7625	MIR1234	microRNA 1234
102465493	0.75675676	MIR6821	microRNA 6821
102465431	0.75641026	MIR6722	microRNA 6722
102464835	0.75510204	NA	NA
102466982	0.75384615	MIR6729	microRNA 6729
693235	0.75229358	MIR92B	microRNA 92b
198437	0.75204082	LKAAEAR1	LKAAEAR motif containing 1
100423014	0.75	MIR3196	microRNA 3196
102465836	0.75	MIR7846	microRNA 7846
100616340	0.74647887	MIR4758	microRNA 4758
102465802	0.74576271	MIR7704	microRNA 7704
100126351	0.74390244	MIR939	microRNA 939
100616187	0.74390244	MIR4783	microRNA 4783

bioRxiv preprint doi: https://doi.org/10.1101/2023.03.30.534966; this version posted March 31, 2023. The copyright holder for this preprint (which was not certified by peer review) is the author/funder, who has granted bioRxiv a license to display the preprint in perpetuity. It is made available under aCC-BY 4.0 International license.

57801	0.74318745	HES4	hes family bHLH transcription factor 4
494324	0.74107143	MIR375	microRNA 375
128408	0.73850316	BHLHE23	basic helix-loop-helix family member e23
101926933	0.7384077	LOC101926933	uncharacterized LOC101926933
474383	0.73835125	F8A2	coagulation factor VIII associated 2
163732	0.73480243	CITED4	Cbp/p300 interacting transactivator with Glu/Asp rich carboxy-terminal domain 4
92304	0.73157163	SCGB3A1	secretoglobin family 3A member 1
102465517	0.73134328	MIR6858	microRNA 6858
100616448	0.7297293	NA	NA
442907	0.72972973	MIR339	microRNA 339
100500811	0.72941176	MIR3621	microRNA 3621
2848	0.72744015	GPR25	G protein-coupled receptor 25
102465835	0.72727273	MIR7845	microRNA 7845
389813	0.7266925	AJM1	apical junction component 1 homolog
100423023	0.7260274	MIR3197	microRNA 3197
102465524	0.72580645	MIR6869	microRNA 6869
64975	0.72439204	MRPL41	mitochondrial ribosomal protein L41
102465435	0.72307692	MIR6727	microRNA 6727
100616115	0.72151899	MIR4469	microRNA 4469
100616342	0.72058824	MIR4638	microRNA 4638
5020	0.71937639	OXT	oxytocin/neurophysin I prepropeptide
406941	0.71910112	MIR149	microRNA 149
8511	0.71856608	MMP23A	matrix metallopeptidase 23A (pseudogene)
102464823	0.71666667	MIR6068	microRNA 6068
122748	0.71641791	OR11H6	olfactory receptor family 11 subfamily H member 6
10419	0.71604938	PRMT5	protein arginine methyltransferase 5
101929634	0.71428571	LINC02280	long intergenic non-protein coding RNA 2280
101927953	0.71405094	C5orf66-AS1	C5orf66 antisense RNA 1
345456	0.71320755	PFN3	profilin 3
389199	0.71296296	LOC389199	uncharacterized LOC389199
100302137	0.7125	MIR1914	microRNA 1914
440836	0.71229537	ODF3B	outer dense fiber of sperm tails 3B
100302138	0.71212121	MIR1292	microRNA 1292
102466199	0.71212121	MIR6825	microRNA 6825
100616242	0.71186441	MIR4673	microRNA 4673
79144	0.71072151	PPDPF	pancreatic progenitor cell differentiation and proliferation factor
724028	0.71	MIR658	microRNA 658
100500808	0.70967742	MIR3917	microRNA 3917
100500810	0.70886076	MIR3620	microRNA 3620
100506681	0.70731707	JARID2-AS1	JARID2 antisense RNA 1
54345	0.70679612	SOX18	SRY-box 18
2928	0.70667447	GSC2	goosecoid homeobox 2
100616465	0.70666667	MIR4656	microRNA 4656
643965	0.7060241	TMEM88B	transmembrane protein 88B

bioRxiv preprint doi: https://doi.org/10.1101/2023.03.30.534966; this version posted March 31, 2023. The copyright holder for this preprint (which was not certified by peer review) is the author/funder, who has granted bioRxiv a license to display the preprint in perpetuity. It is made available under aCC-BY 4.0 International license.

100187716	0.70588235	MIR1224	microRNA 1224
168620	0.70538244	BHLHA15	basic helix-loop-helix family member a15
100422893	0.70535714	MIR3154	microRNA 3154
100616438	0.70491803	MIR4632	microRNA 4632
102465434	0.70491803	MIR6726	microRNA 6726
102465491	0.70491803	MIR6819	microRNA 6819
4857	0.70442992	NOVA1	NOVA alternative splicing regulator 1
100313824	0.70434783	MIR663B	microRNA 663b
100616480	0.70422535	MIR4479	microRNA 4479
7293	0.70387487	TNFRSF4	TNF receptor superfamily member 4
94234	0.703207	FOXQ1	forkhead box Q1
643988	0.70311751	FNDC10	fibronectin type III domain containing 10
100616120	0.7027027	MIR4695	microRNA 4695
100847072	0.70212766	MIR5008	microRNA 5008
55194	0.7019774	EVA1B	eva-1 homolog B
100422860	0.70149254	MIR4292	microRNA 4292
55502	0.70075125	HES6	hes family bHLH transcription factor 6
1052	0.70018365	CEBPD	CCAAT enhancer binding protein delta
100126339	0.6996904	MIR941-2	microRNA 941-2
100313887	0.69892473	MIR2277	microRNA 2277
140701	0.69885983	ABHD16B	abhydrolase domain containing 16B
100422889	0.69863014	MIR3194	microRNA 3194
724031	0.69662921	MIR661	microRNA 661
152	0.69516205	ADRA2C	adrenoceptor alpha 2C
415116	0.69506726	PIM3	Pim-3 proto-oncogene, serine/threonine kinase
102466740	0.69491525	MIR6808	microRNA 6808
56731	0.69465021	SLC2A4RG	SLC2A4 regulator
442425	0.69438029	FOXB2	forkhead box B2
345630	0.69387755	FBLL1	fibrillarin like 1
693187	0.69387755	MIR602	microRNA 602
102465526	0.69354839	MIR6872	microRNA 6872
388581	0.69347674	C1QTNF12	C1q and TNF related 12
401115	0.69211325	C4orf48	chromosome 4 open reading frame 48
1365	0.69152276	CLDN3	claudin 3
100126329	0.69021739	MIR941-1	microRNA 941-1
100847037	0.69	MIR5001	microRNA 5001
54729	0.69	NKX1-1	NK1 homeobox 1
54461	0.68958236	FBXW5	F-box and WD repeat domain containing 5
100616160	0.68918919	MIR4655	microRNA 4655
100422950	0.68852459	MIR3141	microRNA 3141
102466223	0.68852459	MIR7114	microRNA 7114
388849	0.68776052	CCDC188	coiled-coil domain containing 188
100616346	0.6875	MIR4649	microRNA 4649
9022	0.68741355	CLIC3	chloride intracellular channel 3
29952	0.68701467	DPP7	dipeptidyl peptidase 7
7349	0.68642447	UCN	urocortin

bioRxiv preprint doi: https://doi.org/10.1101/2023.03.30.534966; this version posted March 31, 2023. The copyright holder for this preprint (which was not certified by peer review) is the author/funder, who has granted bioRxiv a license to display the preprint in perpetuity. It is made available under aCC-BY 4.0 International license.

2875	0.68443598	GPT	glutamic--pyruvic transaminase
8326	0.68434559	FZD9	frizzled class receptor 9
389816	0.68422684	LRRC26	leucine rich repeat containing 26
102464824	0.6835443	MIR6069	microRNA 6069
94032	0.68280764	CAMK2N2	calcium/calmodulin dependent protein kinase II inhibitor 2
7425	0.68265802	VGF	VGF nerve growth factor inducible
2301	0.68248359	FOXE3	forkhead box E3
102465490	0.68181818	MIR6816	microRNA 6816
100423020	0.68131868	MIR4258	microRNA 4258
102466271	0.68115942	MIR6893	microRNA 6893
8971	0.68044417	H1FX	H1 histone family member X
729627	0.6803995	PRR23A	proline rich 23A
101927062	0.68037135	LINC02294	long intergenic non-protein coding RNA 2294
4582	0.68010715	MUC1	mucin 1, cell surface associated
551	0.67957584	AVP	arginine vasopressin
163933	0.67923795	FAM43B	family with sequence similarity 43 member B
414919	0.67882353	C8orf82	chromosome 8 open reading frame 82
339451	0.6786898	KLHL17	kelch like family member 17
728229	0.67821068	TMEM191B	transmembrane protein 191B
100169751	0.6781923	RNA5S1	RNA, 5S ribosomal 1
84808	0.67764877	PERM1	PPARGC1 and ESRR induced regulator, muscle 1
100422927	0.67692308	MIR4291	microRNA 4291
54863	0.67688535	TOR4A	torsin family 4 member A
100316904	0.67671061	SAP25	Sin3A associated protein 25
83590	0.67612485	TMUB1	transmembrane and ubiquitin like domain containing 1
165257	0.67585946	C1QL2	complement C1q like 2
92070	0.67567568	CTBP1-DT	CTBP1 divergent transcript
150223	0.67560729	YDJC	YdjC chitooligosaccharide deacetylase homolog
10227	0.67550593	MFSD10	major facilitator superfamily domain containing 10
6576	0.67536855	SLC25A1	solute carrier family 25 member 1
402635	0.67528035	GRIFIN	galectin-related inter-fiber protein
353137	0.67507003	LCE1F	late cornified envelope 1F
8784	0.67462312	TNFRSF18	TNF receptor superfamily member 18
339456	0.67458432	TMEM52	transmembrane protein 52
377841	0.67435932	ENTPD8	ectonucleoside triphosphate diphosphohydrolase 8
3083	0.67433303	HGFAC	HGF activator
83481	0.6742822	EPPK1	epiplakin 1
653604	0.67396594	HIST2H3D	histone cluster 2 H3 family member d
51162	0.6730811	EGFL7	EGF like domain multiple 7
101928767	0.67307692	LINC02328	long intergenic non-protein coding RNA 2328
116983	0.67297203	ACAP3	ArfGAP with coiled-coil, ankyrin repeat and PH domains 3
441478	0.67288136	NRARP	NOTCH regulated ankyrin repeat protein
100423027	0.67272727	MIR4266	microRNA 4266

bioRxiv preprint doi: https://doi.org/10.1101/2023.03.30.534966; this version posted March 31, 2023. The copyright holder for this preprint (which was not certified by peer review) is the author/funder, who has granted bioRxiv a license to display the preprint in perpetuity. It is made available under aCC-BY 4.0 International license.

340260	0.67225748	UNCX	UNC homeobox
102466748	0.67213115	MIR6845	microRNA 6845
642658	0.67169615	SCX	scleraxis bHLH transcription factor
102465977	0.67164179	MIR6829	microRNA 6829
100526833	0.67152125	SEPT5-GP1BB	SEPT5-GP1BB readthrough
91289	0.67151592	LMF2	lipase maturation factor 2
102465511	0.67142857	MIR6848	microRNA 6848
221908	0.67133956	PPP1R35	protein phosphatase 1 regulatory subunit 35
9997	0.67130919	SCO2	SCO2, cytochrome c oxidase assembly protein
386685	0.67124857	KRTAP10-12	keratin associated protein 10-12
4738	0.67124071	NEDD8	neural precursor cell expressed, developmentally down-regulated 8
647219	0.67112676	ASCL5	achaete-scute family bHLH transcription factor 5
4056	0.67091454	LTC4S	leukotriene C4 synthase
100423011	0.67073171	MIR3138	microRNA 3138
440829	0.67031678	SHISA8	shisa family member 8
100126332	0.67021277	MIR943	microRNA 943
3754	0.67016164	KCNF1	potassium voltage-gated channel modifier subfamily F member 1
389332	0.67006599	SMIM32	small integral membrane protein 32
113655	0.66962617	MFSD3	major facilitator superfamily domain containing 3
54998	0.66874292	AURKAIP1	aurora kinase A interacting protein 1
727800	0.66852368	RNF208	ring finger protein 208
142680	0.66850638	SLC34A3	solute carrier family 34 member 3
25907	0.66850524	TMEM158	transmembrane protein 158 (gene/pseudogene)
102724312	0.66840149	LINC01770	long intergenic non-protein coding RNA 1770
54905	0.66811549	CYP2W1	cytochrome P450 family 2 subfamily W member 1
30012	0.66783585	TLX3	T cell leukemia homeobox 3
83482	0.66783278	SCRT1	scratch family transcriptional repressor 1
402682	0.66767068	UFSP1	UFM1 specific peptidase 1 (inactive)
126789	0.66763099	PUSL1	pseudouridylate synthase-like 1
23237	0.66754156	ARC	activity regulated cytoskeleton associated protein
83756	0.66741798	TAS1R3	taste 1 receptor member 3
53834	0.66729712	FGFRL1	fibroblast growth factor receptor like 1
7012	0.66728281	TERC	telomerase RNA component
2907	0.66676593	GRINA	glutamate ionotropic receptor NMDA type subunit associated protein 1
100302156	0.66666667	MIR1229	microRNA 1229
100302237	0.66666667	MIR1281	microRNA 1281
100422939	0.66666667	MIR3147	microRNA 3147
100616178	0.66666667	MIR4641	microRNA 4641
100616353	0.66666667	NA	NA
100616367	0.66666667	MIR4467	microRNA 4467
102465438	0.66666667	MIR6732	microRNA 6732
693232	0.66666667	MIR647	microRNA 647
20	0.66662058	ABCA2	ATP binding cassette subfamily A member 2

bioRxiv preprint doi: https://doi.org/10.1101/2023.03.30.534966; this version posted March 31, 2023. The copyright holder for this preprint (which was not certified by peer review) is the author/funder, who has granted bioRxiv a license to display the preprint in perpetuity. It is made available under aCC-BY 4.0 International license.

386676	0.66609735	KRTAP10-9	keratin associated protein 10-9
407003	0.66555851	MIR219A2	microRNA 219a-2
441869	0.66507463	ANKRD65	ankyrin repeat domain 65
170487	0.66469428	ACTL10	actin like 10
11219	0.66459276	TREX2	three prime repair exonuclease 2
26233	0.66447035	FBXL6	F-box and leucine rich repeat protein 6
389058	0.66422287	SP5	Sp5 transcription factor
100288152	0.66419919	SLC9A3-AS1	SLC9A3 antisense RNA 1
84968	0.66417028	PNMA6A	PNMA family member 6A
102577424	0.66416792	LINC01574	long intergenic non-protein coding RNA 1574
142678	0.66405633	MIB2	mindbomb E3 ubiquitin protein ligase 2
347454	0.66365979	SOWAHD	sosondowah ankyrin repeat domain family member D
6754	0.66362999	SSTR4	somatostatin receptor 4
64856	0.66355024	VWA1	von Willebrand factor A domain containing 1
386677	0.66337449	KRTAP10-1	keratin associated protein 10-1
100191040	0.6630157	C2CD4D	C2 calcium dependent domain containing 4D
158056	0.66277772	MAMDC4	MAM domain containing 4
100302259	0.6626506	MIR1202	microRNA 1202
1855	0.66245115	DVL1	dishevelled segment polarity protein 1
10912	0.66234608	GADD45G	growth arrest and DNA damage inducible gamma
100126350	0.66233766	MIR933	microRNA 933
29085	0.66232639	PHPT1	phosphohistidine phosphatase 1
386679	0.66231506	KRTAP10-2	keratin associated protein 10-2
100500835	0.66225166	MIR3907	microRNA 3907
1059	0.66197183	CENPB	centromere protein B
1051	0.66155419	CEBPB	CCAAT enhancer binding protein beta
100500913	0.66153846	MIR3714	microRNA 3714
102465507	0.66153846	MIR6842	microRNA 6842
8941	0.66148325	CDK5R2	cyclin dependent kinase 5 regulatory subunit 2
56033	0.66142362	BARX1	BARX homeobox 1
8484	0.66130558	GALR3	galanin receptor 3
5365	0.66127648	PLXNB3	plexin B3
646960	0.66121051	PRSS56	serine protease 56
11255	0.6611929	HRH3	histamine receptor H3
85441	0.66076514	HELZ2	helicase with zinc finger 2
26086	0.6605042	GPSM1	G protein signaling modulator 1
339983	0.66036751	NAT8L	N-acetyltransferase 8 like
339453	0.66022727	TMEM240	transmembrane protein 240
100419743	0.66012489	DBET	D4Z4 binding element transcript (non-protein coding)
389333	0.66009371	PROB1	proline rich basic protein 1
148398	0.659933	SAMD11	sterile alpha motif domain containing 11
79581	0.6597086	SLC52A2	solute carrier family 52 member 2
390992	0.65969719	HES3	hes family bHLH transcription factor 3
441476	0.65946919	STPG3	sperm-tail PG-rich repeat containing 3

bioRxiv preprint doi: https://doi.org/10.1101/2023.03.30.534966; this version posted March 31, 2023. The copyright holder for this preprint (which was not certified by peer review) is the author/funder, who has granted bioRxiv a license to display the preprint in perpetuity. It is made available under aCC-BY 4.0 International license.

100422826	0.65934066	MIR4274	microRNA 4274
6339	0.65930654	SCNN1D	sodium channel epithelial 1 delta subunit
26873	0.65926633	OPLAH	5-oxoprolinase, ATP-hydrolysing
101928158	0.65920826	LAMA5-AS1	LAMA5 antisense RNA 1
643596	0.65920608	RNF224	ring finger protein 224
386680	0.65913043	KRTAP10-5	keratin associated protein 10-5
2783	0.65910702	GNB2	G protein subunit beta 2
8636	0.65893271	SSNA1	SS nuclear autoantigen 1
5413	0.65887534	5-Sep	septin 5
80139	0.65886209	ZNF703	zinc finger protein 703
386681	0.6586758	KRTAP10-8	keratin associated protein 10-8
56654	0.65859816	NPDC1	neural proliferation, differentiation and control 1
100422994	0.65853659	MIR4267	microRNA 4267
644524	0.65852742	NKX2-4	NK2 homeobox 4
2261	0.65822947	FGFR3	fibroblast growth factor receptor 3
677766	0.65807963	SCARNA2	small Cajal body-specific RNA 2
100130449	0.65794162	LOC100130449	uncharacterized LOC100130449
100423028	0.65789474	MIR4254	microRNA 4254
391723	0.65784114	HELT	helt bHLH transcription factor
100422928	0.65765766	MIR3127	microRNA 3127
100126348	0.65740741	MIR760	microRNA 760
401357	0.65740741	LOC401357	uncharacterized LOC401357
954	0.65736382	ENTPD2	ectonucleoside triphosphate diphosphohydrolase 2
118442	0.65723414	GPR62	G protein-coupled receptor 62
375790	0.65710079	AGRN	agrin
158062	0.65628761	LCN6	lipocalin 6
693159	0.65625	MIR574	microRNA 574
8022	0.65586907	LHX3	LIM homeobox 3
102465536	0.6557377	MIR6890	microRNA 6890
4636	0.65542938	MYL5	myosin light chain 5
389458	0.65514104	RBAKDN	RBAK downstream neighbor (non-protein coding)
9401	0.65507777	RECQL4	RecQ like helicase 4
3425	0.65448315	IDUA	iduronidase, alpha-L-
102060282	0.65443038	RASSF1-AS1	RASSF1 antisense RNA 1
401934	0.65439856	RNF223	ring finger protein 223
100616447	0.65432099	MIR4780	microRNA 4780
255374	0.65429363	MBLAC1	metallo-beta-lactamase domain containing 1
386674	0.6542811	KRTAP10-6	keratin associated protein 10-6
5600	0.65386251	MAPK11	mitogen-activated protein kinase 11
100129722	0.65385788	STPG3-AS1	STPG3 antisense RNA 1
90120	0.65351389	TMEM250	transmembrane protein 250
386684	0.65324385	KRTAP12-4	keratin associated protein 12-4
54587	0.65298713	MXRA8	matrix remodeling associated 8
733	0.6529786	C8G	complement C8 gamma chain
100286938	0.65294592	VPS13A-AS1	VPS13A antisense RNA 1
386682	0.65293512	KRTAP10-3	keratin associated protein 10-3

bioRxiv preprint doi: https://doi.org/10.1101/2023.03.30.534966; this version posted March 31, 2023. The copyright holder for this preprint (which was not certified by peer review) is the author/funder, who has granted bioRxiv a license to display the preprint in perpetuity. It is made available under aCC-BY 4.0 International license.

51052	0.65282392	PRLH	prolactin releasing hormone
80772	0.65258329	CPTP	ceramide-1-phosphate transfer protein
84179	0.65243662	SLC49A3	solute carrier family 49 member 3
26012	0.6521887	NSMF	NMDA receptor synaptonuclear signaling and neuronal migration factor
286256	0.65209349	LCN12	lipocalin 12
100616312	0.65116279	MIR4478	microRNA 4478
5730	0.65111587	PTGDS	prostaglandin D2 synthase
1961	0.6510623	EGR4	early growth response 4
100422975	0.65079365	MIR4252	microRNA 4252
102466270	0.65079365	MIR6741	microRNA 6741
137797	0.65073375	LYPD2	LY6/PLAUR domain containing 2
100500828	0.65060241	MIR3619	microRNA 3619
114822	0.65000651	RHPN1	rhophilin Rho GTPase binding protein 1

* The exonic and intronic regions of the human reference genome hgp38 were searched with a criterion set at a GC content of between 65 and 100%, as detailed in Materials and Methods. NA: not annotated.

s

bioRxiv preprint doi: https://doi.org/10.1101/2023.03.30.534966; this version posted March 31, 2023. The copyright holder for this preprint (which was not certified by peer review) is the author/funder, who has granted bioRxiv a license to display the preprint in perpetuity. It is made available under aCC-BY 4.0 International license.

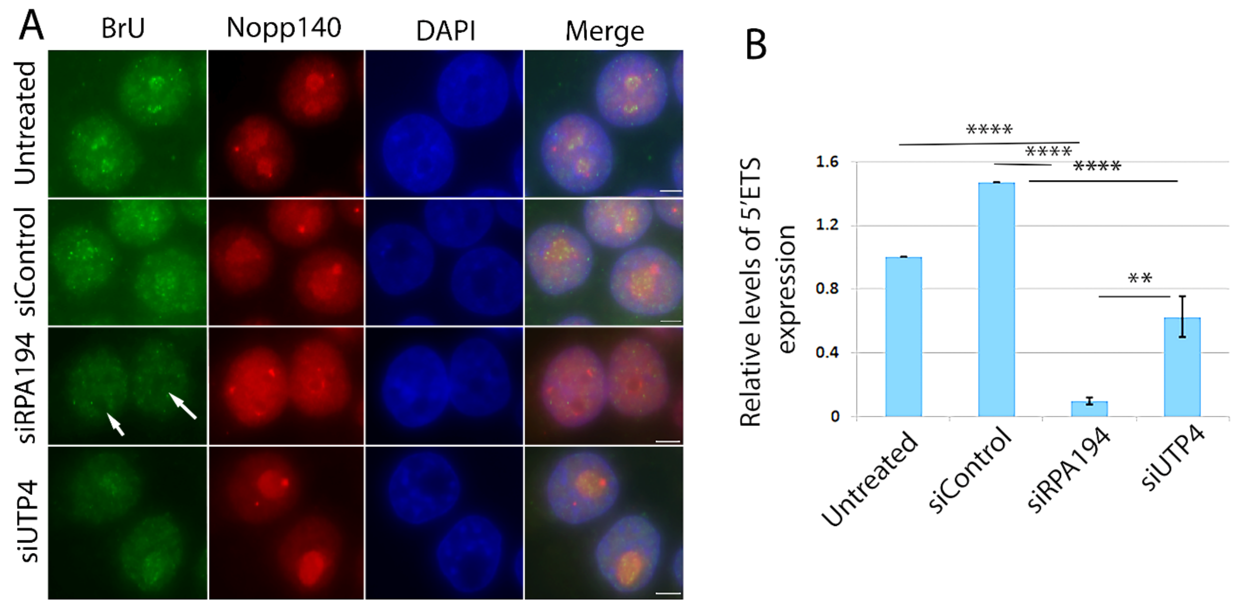

Suppl. Figure 1: The transcription of rDNA was significantly reduced in cells treated with siRPA194 and to a lesser extent with siUTP4. (A) BrU pulse labeling for 5 minutes showed a significant loss of BrU signal in siRPA194 treated cells (A, arrows), compared to controls where more intense labeling was detected in nucleoli. (B) Quantitative RT-PCR showed a significant reduction in 5'ETS RNA expression in siRPA194 and siUTP4 treated cells. Bar= 5 mm. ****p<0.001, **p<0.01

bioRxiv preprint doi: https://doi.org/10.1101/2023.03.30.534966; this version posted March 31, 2023. The copyright holder for this preprint (which was not certified by peer review) is the author/funder, who has granted bioRxiv a license to display the preprint in perpetuity. It is made available under aCC-BY 4.0 International license.

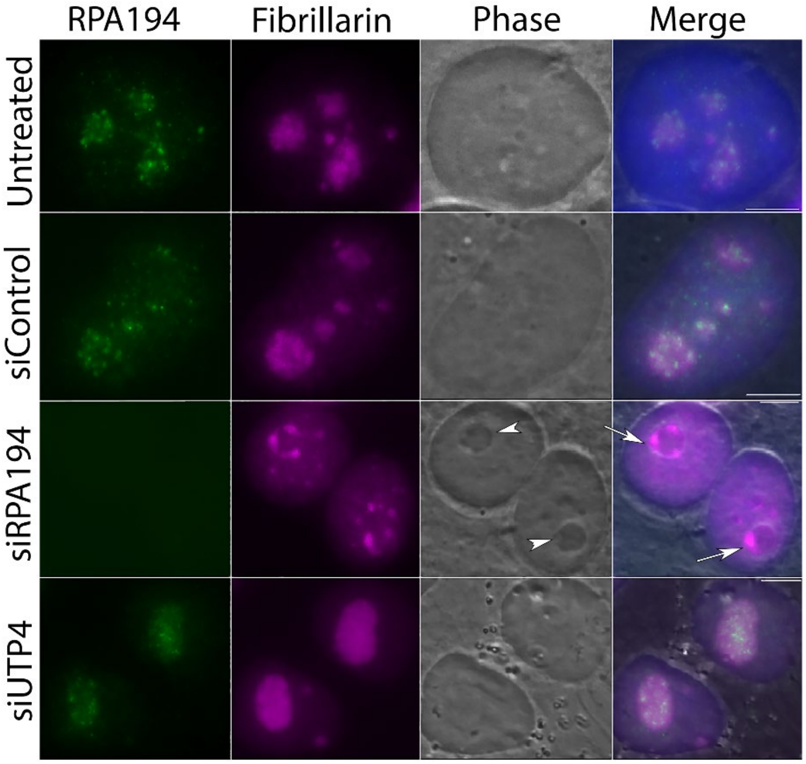

Suppl. Figure 2: siRPA194, but not siUTP4 treated HeLa cells, lost the RPA194 signal (green). Nucleolar segregation was detected by the immunolabeling of fibrillarin (magenta). Cap-like structures enriched with firbrillar components (arrows) juxtaposed the reminants of the nucleoli primarily containing granular components (arrowheads). Bars= 5 mm

bioRxiv preprint doi: https://doi.org/10.1101/2023.03.30.534966; this version posted March 31, 2023. The copyright holder for this preprint (which was not certified by peer review) is the author/funder, who has granted bioRxiv a license to display the preprint in perpetuity. It is made available under aCC-BY 4.0 International license.

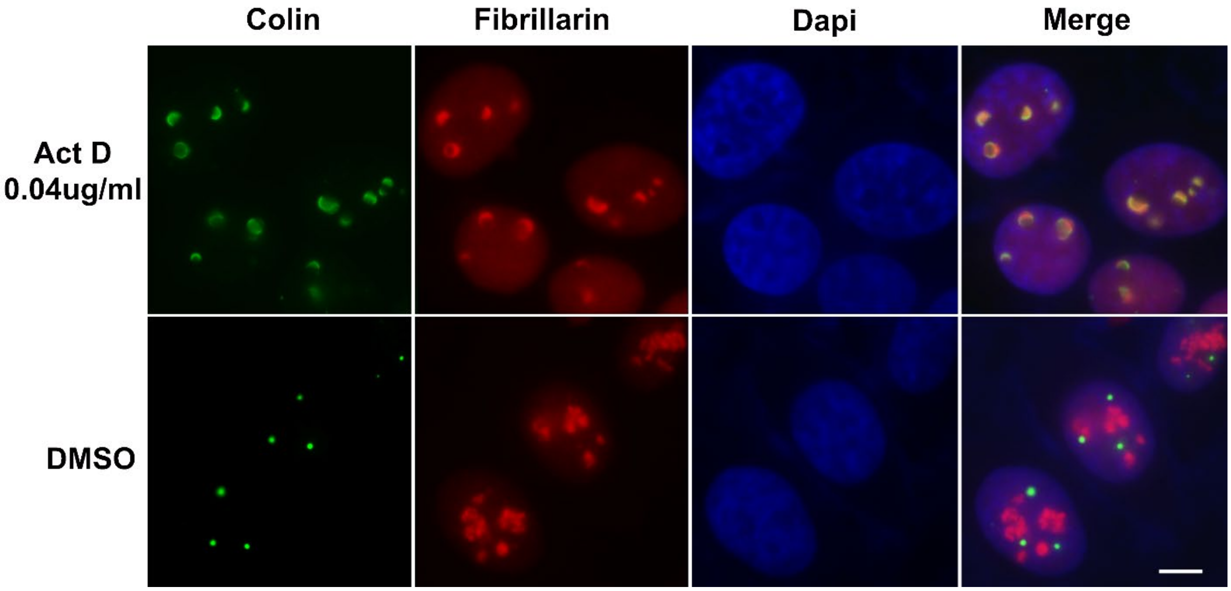

Suppl. Figure 3: Treatment with a low concentration (0.04ug/ml) of actinomycin D (Act D) induces nucleolar segregations and disruption of Cajal bodies as detected by anti-fibrillarin antibodies (red) and anti-coilin (green) antibodies. Bar= 5 mm

bioRxiv preprint doi: https://doi.org/10.1101/2023.03.30.534966; this version posted March 31, 2023. The copyright holder for this preprint (which was not certified by peer review) is the author/funder, who has granted bioRxiv a license to display the preprint in perpetuity. It is made available under aCC-BY 4.0 International license.

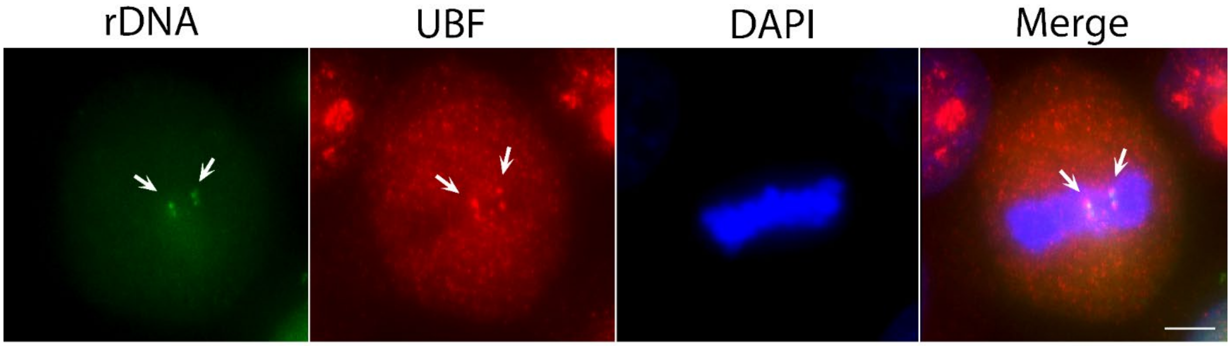

Suppl. Figure 4: The CRISPR-Cas9 sgRNA labeling of rDNA was validated using mitotic cells where UBF was found to be colocalized with the labeling (arrows), confirming that the sgRNA sets indeed labeled rDNA chromatin on mitotic chromosomes (arrow). Bar= 5 mm

bioRxiv preprint doi: https://doi.org/10.1101/2023.03.30.534966; this version posted March 31, 2023. The copyright holder for this preprint (which was not certified by peer review) is the author/funder, who has granted bioRxiv a license to display the preprint in perpetuity. It is made available under aCC-BY 4.0 International license.

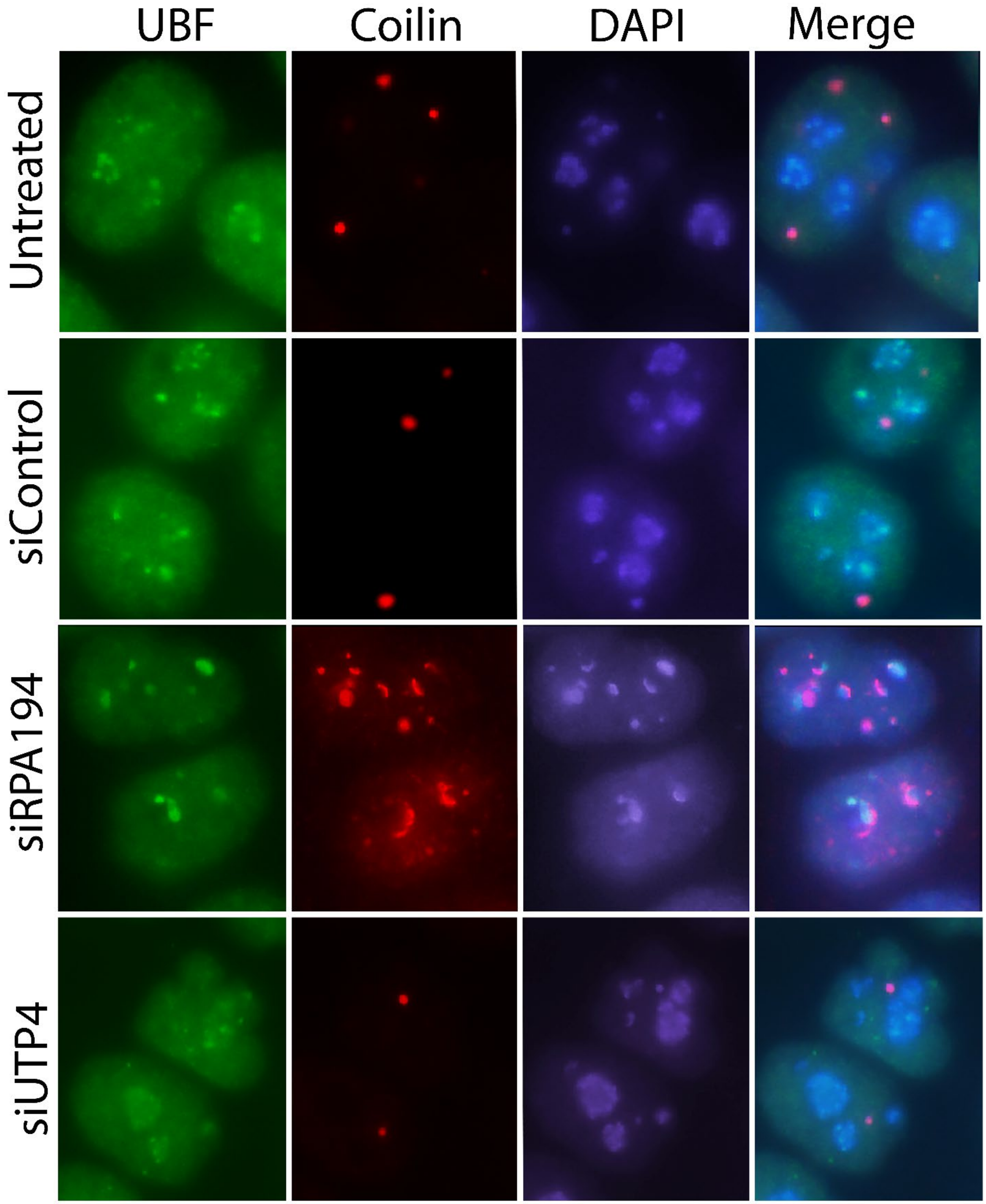

Suppl. Fig. 5: Cajal bodies are significantly altered in siRPA194 treated cells. Cells were co-immunolabeled with antibodies against UBF and coilin. Bar= 5 mm

bioRxiv preprint doi: https://doi.org/10.1101/2023.03.30.534966; this version posted March 31, 2023. The copyright holder for this preprint (which was not certified by peer review) is the author/funder, who has granted bioRxiv a license to display the preprint in perpetuity. It is made available under aCC-BY 4.0 International license.

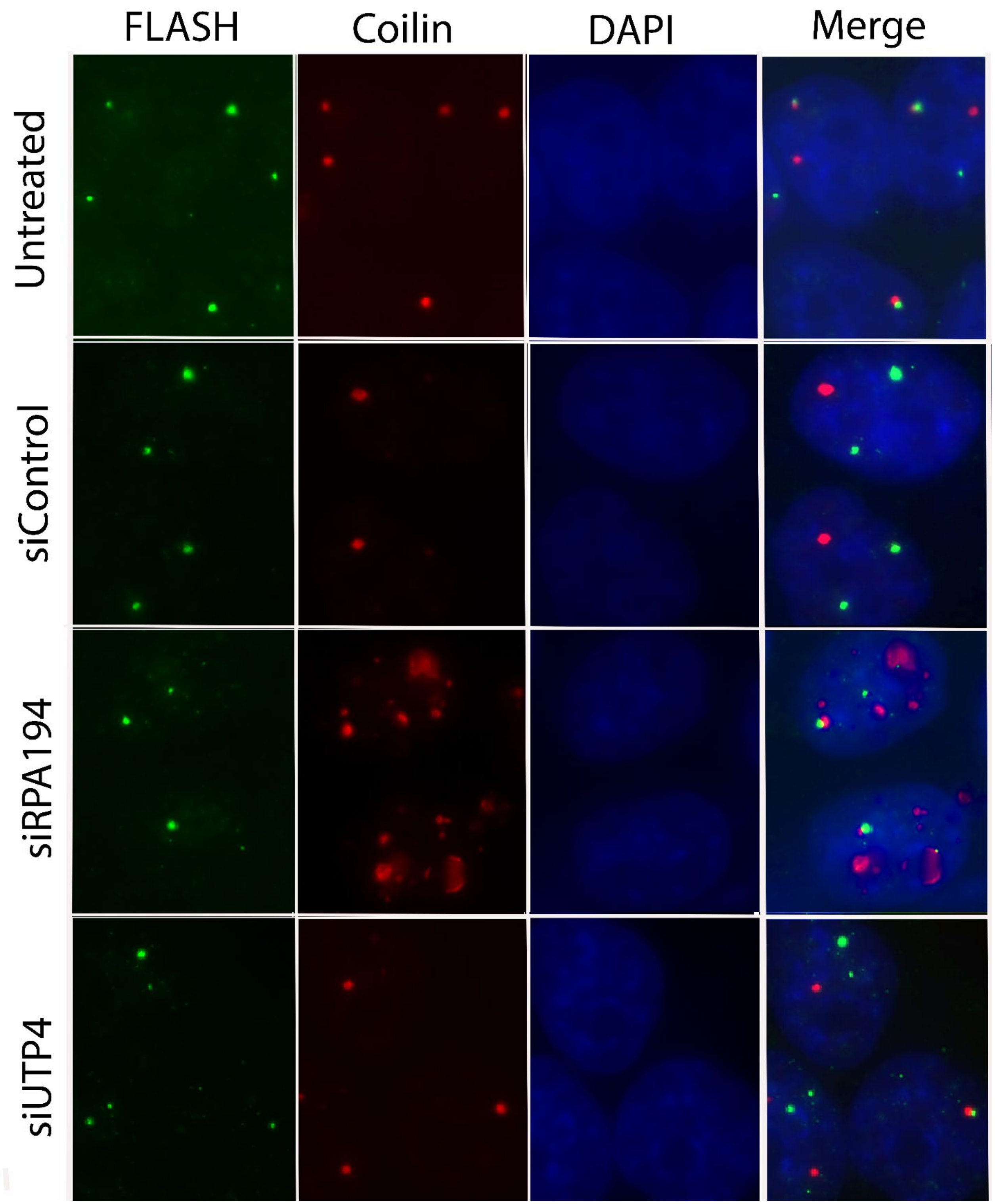

Suppl. Figure 6: While Cajal bodies change significantly in siRPA194 treated cells, histone locus bodies do not show detectable changes. Bar= 5 mm
